# Biodegradable temporizing matrix (BTM): A valuable reconstructive adjunct

**DOI:** 10.1016/j.jdcr.2025.10.079

**Published:** 2026-01-21

**Authors:** Amy Long, Benvon Moran, Patrick Ormond, Jennifer Boggs

**Affiliations:** Department of Dermatology, St James’s Hospital, Dublin, Ireland

**Keywords:** skin cancer, surgery, wound management

## Introduction

Biodegradable temporizing matrix (BTM; PolyNovo Biomaterials Pty Ltd, Melbourne, Australia) has emerged as a valuable adjunct in wound care. This synthetic polyurethane dermal substitute is designed for use on complex wounds, such as those resulting from burns, trauma, and infection.^1^ Typically, BTM is applied in a two-stage process: initial placement of the matrix, followed by removal of the sealing membrane and application of a split-thickness skin graft to the neodermis.^2^^,^^3^ While its use in reconstructive surgery has been described, including isolated cases following Mohs surgery,^4^ its application by dermatologists has not previously been reported. We present a novel application of BTM to support secondary intention healing (SIH) in dermatologic surgery.

## Case series

Between March and June 2024, 7 patients with complex postsurgical defects were treated with BTM at a tertiary referral centre. BTM was chosen due to technical considerations such as risk of alar rim retraction, defects spanning multiple facial subunits, or deep wounds where immediate full-thickness skin grafting was unfavorable. Patient-related factors, such as the need for a simplified wound care regimen, also informed our decision.

## Case 1

**Fig 1:**
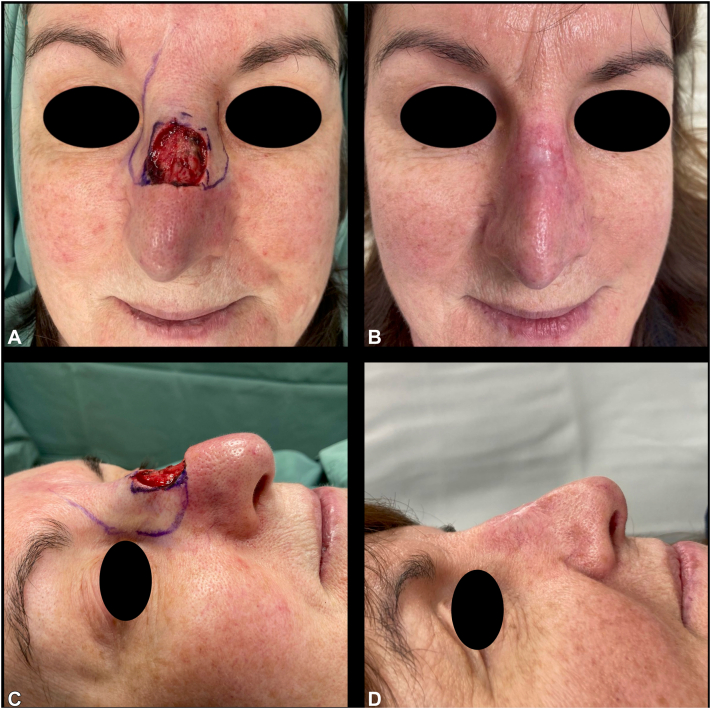
Case 1, before **(A** and **C)** BTM placement, and 12 weeks postoperatively **(B** and **D)**. *BTM*, Biodegradable temporizing matrix.

A 57-year-old female underwent Mohs micrographic surgery (MMS) for an infiltrative basal cell carcinoma (BCC) (10 × 10 mm) on the nasal dorsum. Three Mohs layers were taken to achieve tumor extirpation, which resulted in a deep defect measuring 25 × 25 mm. A previous rhinoplasty had reduced little tissue laxity, limiting tissue movement for a local flap reconstruction. An immediate full-thickness skin graft (FTSG) would not restore volume and lead to a suboptimal cosmetic outcome. BTM was applied with the intention of doing a delayed FTSG in the subsequent weeks. One week postoperatively, erythema raised concern for infection; bacterial swabs grew *Staphylococcus aureus.* She was treated with flucloxacillin with good response. By week 5, the wound had healed with excellent volume and contour, negating the need for a FTSG. Her cosmetic outcome was excellent, see Fig 1.

## Case 2

**Fig 2:**
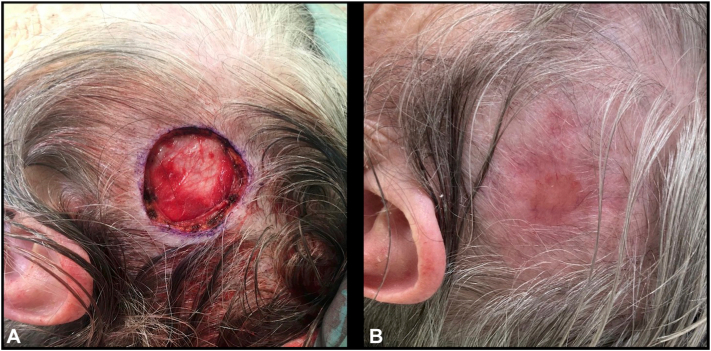
Case 2, before **(A)** BTM placement, and 14 weeks postoperatively **(B)**. *BTM*, Biodegradable temporizing matrix.

A 79-year-old female underwent standard surgical excision of a nodular BCC on the left parietal scalp. BTM was chosen to simplify wound care, as the patient was elderly, lived alone, and could not see or manage the wound herself. A wound infection developed at 2 weeks postoperatively; bacterial swabs confirmed *Staphylococcus aureus*, and she responded well to flucloxacillin. Her outcome was excellent, see Fig 2.

**Fig 3:**
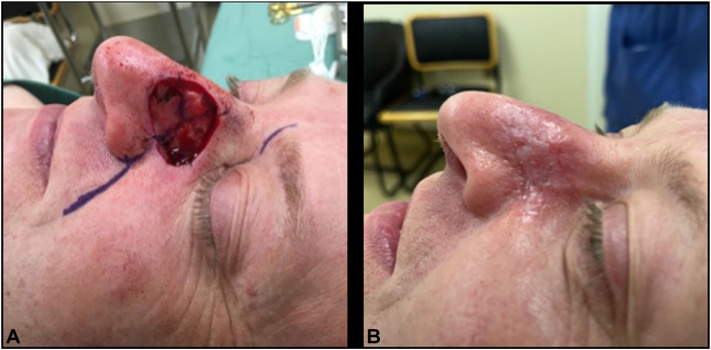
Case 4, before **(A)** BTM placement, and 12 weeks postoperatively **(B)**. BTM, Biodegradable temporizing matrix.

## Case 3

An 86-year-old male presented with 3 nodular BCCs on the right nose: nasal sidewall (6 × 4 mm), superior ala (size 8 × 4 mm), and inferior ala (5 × 5 mm). Mohs micrographic surgery was performed, requiring 4 layers to achieve tumor clearance at the superior ala, and 2 layers at the other sites. The resulting defects coalesced to form 1 large defect (25 × 14 mm) involving multiple facial subunits. The risk of alar rim retraction was significant if SIH was chosen. A cheek advancement flap was designed to reconstruct the superior aspect of the defect, while BTM was applied to the inferior defect. No postoperative complications were observed.

## Case 4

A 51-year-old male underwent MMS for a nodular BCC (22 × 20 mm) on the left nasal sidewall. Two Mohs layers were needed, resulting in a 24 × 26 mm defect, see Fig 3. The patient’s young age and preserved skin elasticity limited repair options. An immediate FTSG was avoided due to poor color and textural match at potential donor sites. For these reasons, BTM was chosen to facilitate SIH. No complications occurred.

## Case 5

A 62-year-old female had MMS for a nodular BCC (7 × 7 mm) on the right nasal ala. Two Mohs layers resulted in a 9 × 8 mm defect. BTM was selected to prevent alar rim retraction and because the wound was too deep to permit a satisfactory cosmetic outcome with an immediate FTSG. The postoperative course was uncomplicated.

## Case 6

A 53-year-old female smoker underwent MMS for a nodular BCC (10 × 5 mm) on the left nasal sidewall. Six Mohs layers were required, yielding a large 32 × 29 mm defect spanning 2 facial subunits. Due to the extent of the defect and the patient’s smoking status, a FTSG was deemed unlikely to succeed. BTM was used to facilitate SIH, without complications.

## Case 7

An 83-year-old male had MMS for a nodular BCC (12 × 15 mm) on the right nasal tip. After 2 Mohs layers, a 19 × 16 mm defect lay 1 mm from the alar rim. Given the proximity to the alar rim, SIH would likely cause rim retraction. An immediate FTSG was unfavorable due to the depth of the wound. BTM was applied and the postoperative course was uneventful.

## Discussion

BTM was well tolerated by our patients. Two patients were treated with oral antibiotics for *Staphylococcus aureus* wound infections. We now routinely use bacterial-binding dressings (Sorbact, Essity, Stockholm, Sweden) to reduce infection risk. All wounds healed successfully by secondary intention without the need for subsequent skin grafting.

**Fig 4:**
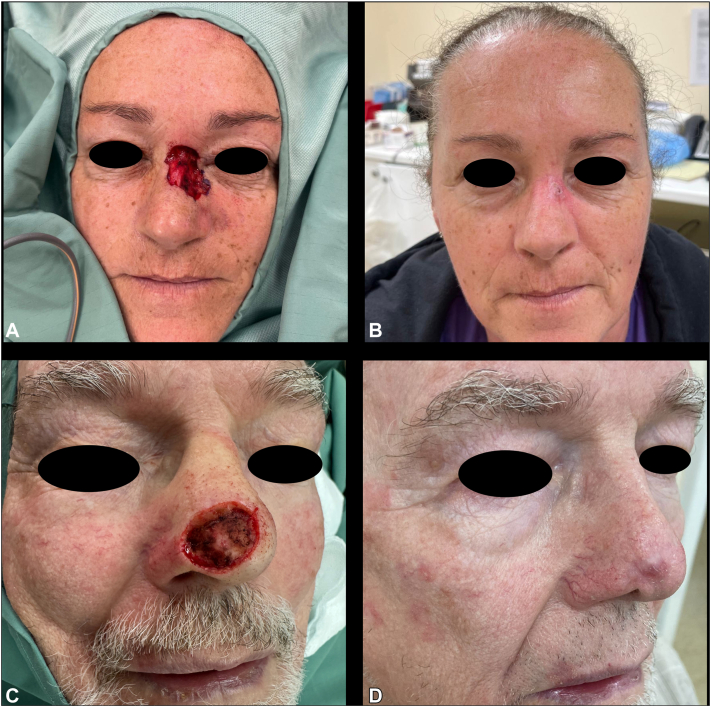
Case 6, before **(A)** BTM placement, and 12 weeks postoperatively **(B)**; and Case 7, before **(C)** BTM placement, and 12 weeks postoperatively **(D)**. *BTM*, Biodegradable temporizing matrix.

Favorable cosmetic and functional outcomes were observed across all cases, as demonstrated in Fig 1, Fig 2, Fig 3, Fig 4. Patients were discharged from the dressing clinic between 4 and 14 weeks postoperatively. Without BTM, SIH typically demands a longer follow-up period, as re-epithelialization takes longer to occur. In our series, patients attended the department weekly for wound review and dressing changes, with no need for at-home wound care—a contrast to traditional SIH dressing management, which often requires both more frequent visits and significant patient involvement.

The use of BTM with secondary intention healing in dermatologic surgery addresses several reconstructive challenges, such as large defect size, limited available adjacent skin and exposure of delicate perichondrium. In appropriately selected cases, BTM with SIH offers a simplified and effective alternative to extensive tissue reconstruction or delayed skin grafting, while also minimizing attendances at dressing clinics. These merits are especially relevant for the growing population of elderly patients. An additional advantage of BTM is cost: at our institution, and as reported elsewhere,^5^ BTM is significantly less expensive than biologic dermal substitutes such as Integra Dermal Regeneration Template (LifeSciences), potentially improving accessibility and resource efficiency. Although the economic benefits have not been formally studied, BTM may offer a cost-effective solution that conserves health care resources without compromising patient care.

## Conclusion

Dermatologic surgery continues to evolve in its precision and scope. This case series highlights the potential of BTM to expand reconstructive options by supporting secondary intention healing—simplifying postoperative care, improving cosmetic and functional outcomes, and reducing the burden on patients and health care systems.

## Conflicts of interest

None disclosed.
